# Rule-based semi-automated method to segment black hole multiple sclerosis lesions on post-gadolinium 2D T1-weighted brain images

**DOI:** 10.1007/s00330-026-12577-6

**Published:** 2026-05-02

**Authors:** Rozemarijn M. Mattiesing, Fleur A. Groeneveld, Iman Brouwer, Ronald A. van Schijndel, Frederik Barkhof, Henk J. M. M. Mutsaerts, Hugo Vrenken

**Affiliations:** 1 MS Center Amsterdam, Radiology and Nuclear Medicine, Vrije Universiteit Amsterdam, Amsterdam Neuroscience, Amsterdam UMC, Amsterdam, The Netherlands; 2 Queen Square Institute of Neurology and Centre for Medical Image Computing, University College London, London, UK

**Keywords:** Brain, Computer-assisted, Image processing, Magnetic resonance imaging, Multiple sclerosis

## Abstract

**Objectives:**

To develop a semi-automated method to segment “black hole” lesions on post-gadolinium 2D T1-weighted images (GdT1) in multiple sclerosis (MS) that follows radiological intensity rules and perform multi-center validation.

**Materials and methods:**

Multi-center spin-echo GdT1 images and accompanying proton-density (PD)/T2-weighted images and manual T2 lesion masks of the REFLEXION study (NCT00813709) of suspected/early MS were used. Briefly, the proposed method segments cortical gray matter (GM) to derive a T1-weighted intensity threshold, which is applied inside co-registered T2 lesion masks to segment black hole lesion voxels. It was optimized on a training set (*N* = 40, 57.5% female, mean age 31.4 ± 8.7 (standard deviation) years), and 274 patients formed the test set (61.3% female, age 31.8 ± 8.4 years). Performance was quantified by the Dice similarity coefficient (DSC) and the intraclass correlation coefficient (ICC) for absolute agreement with manual segmentations. Lesion-wise sensitivity and specificity were calculated.

**Results:**

Optimization resulted in: (1) GM selection as minimally 0.8 total WM plus GM partial volume, masked by MNI cortex; (2) normalized mutual information-driven linear co-registration of T2 to GdT1 images, interpolating T2 lesion masks using trilinear interpolation and 0.6 threshold; (3) mean intensity inside GM mask used as upper intensity threshold. The optimized method had acceptable spatial accuracy (DSC: 0.39 ± 0.26) and good volumetric accuracy (ICC: 0.84, 95% CI [0.72, 0.90]. Lesion-wise sensitivity was 0.91 ± 0.19, and lesion-wise specificity was 0.62 ± 0.22.

**Conclusion:**

The proposed method to semi-automatically segment black holes from post-gadolinium T1-weighted images shows acceptable performance. As a potential aid to radiologists, the method is not recommended to be used entirely without human intervention.

**Key Points:**

***Question*** *T1-hypointense “black hole” lesions reflect disease severity in multiple sclerosis but are not routinely quantified due to a lack of reliable analysis methods.*

***Findings*** *A rule-based semi-automated method for GdT1 “black hole” lesion segmentation was developed and optimized, and then validated in a large unseen multi-center test set.*

***Clinical relevance*** *This method adds quantitative information about GdT1 “black hole” lesions to the radiological assessment of multiple sclerosis disease severity, when false positives are manually removed. This can enhance the characterization of individual patients and advance the understanding of the disease.*

**Graphical Abstract:**

**Figure:**
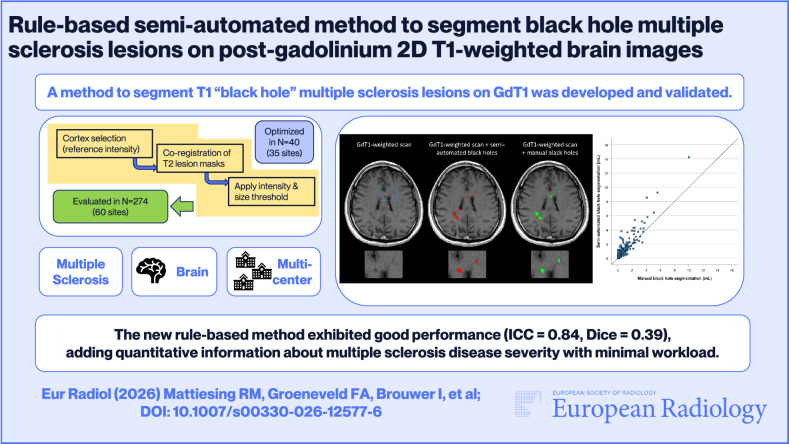

## Introduction

Focal demyelinating lesions in the brain and spinal cord constitute the pathological hallmark of multiple sclerosis (MS) [1, 2]. While all lesions appear hyperintense on T2-weighted MR images, only some are visible as “black holes” (BHs) on T1-weighted images, appearing hypointense compared to surrounding white matter (WM) [3].

BHs correlate with clinical disability at least as well as T2 lesions [4] and mostly represent neuronal tissue destruction and axonal loss [5]. BH lesion counts and volumes are used as secondary endpoints in MS clinical trials [6, 7].

Being able to semi-automatically analyze BHs on 2D T1-weighted images is useful clinically, and there is a lot of existing data (e.g., legacy data from large clinical trials, and current and legacy data from clinical practice) that could provide useful insights and new information about the disease. Several automated methods exist that can detect and/or quantify BHs [8–14]. However, as detailed in Supplementary Table 1, these methods were validated on small groups of subjects from a single center [8–12, 14] or only indirectly without direct comparison to manual labels [9, 13], and the image types necessary for some of these methods are not always available (e.g., (3D-)FLAIR [10, 11] or 3D-T1 [9, 11]).

Therefore, the aim of the current study was to develop and directly validate a (semi)automated method to detect and segment BHs on post-gadolinium (i.e., post-contrast) 2D T1-weighted scans of patients with MS, which is easy to implement and based on rules applied by the radiologist. Post-gadolinium rather than contrast-naive T1-weighted images were chosen because no dataset with expert manual segmentations on contrast-naive T1-weighted images was available. This method was first optimized on a multi-center training set to fine-tune the parameters of the pipeline. Finally, the best performing method was validated on a large multi-center test set with manual labels.

## Materials and methods

### Dataset

Data from the multi-center REFLEXION (REbif FLEXible dosing in early MS extensION; NCT00813709) study [15] was used, which was undertaken in compliance with the Declaration of Helsinki and standards of Good Clinical Practice according to the International Conference on Harmonization of Technical Requirements for Registration of Pharmaceuticals for Human Use. At each center, the relevant institutional review board or independent ethics committee reviewed and approved the study protocols, patient information leaflets, informed consent forms, and investigator brochures. REFLEXION was a preplanned extension of the REFLEX study to assess the longitudinal effects of subcutaneous interferon beta-1a in clinically isolated syndrome and early MS. All patients provided written informed consent to continue follow-up in REFLEXION, which was conducted from 22 December 2008 to 30 August 2013 [15].

### MRI data

We used the 1 × 1 × 3 mm^3^ 2D (dual-echo) PD- and T2-weighted, spin-echo T1-weighted, and post-gadolinium spin-echo T1 (GdT1)-weighted images from the month-60 visit; acquisition details are provided in Supplementary Table 2. The presently defunct Image Analysis Center of Amsterdam UMC (Location VUmc) provided manually corrected brain masks (originally created with the brain extraction tool [16], part of the FMRIB software library (FSL) [17] on contrast-naive T1-weighted images) and manual outlines of T2 lesions (delineated on (dual-echo) proton-density (PD)-weighted images) and T1 (chronic) BHs and enhancing lesions (both of which were delineated on the GdT1-weighted scans). Note that, because in this dataset, the BHs were manually delineated on GdT1-weighted images, we refer to GdT1-weighted images throughout this description, even though contrast-naive T1-weighted images could be used to detect BHs.

From the brain images selected and quality-controlled (QC) for another study [18], we here used only the month-60 visit (*N* = 323, from 64 sites), because there, acute inflammatory edema is expected to be resolved, leaving only chronic BHs.

### Input image quality control

The file integrity of the manual BH lesion overlay files was assessed. Input image quality was then checked visually by a trained expert (R.M.M.) supervised by two senior experts, each with > 20 years of experience (F.B. and H.V.), in the following manner. Image contrast, noise, and artefacts were assessed visually on GdT1 images by R.M.M. in regular consultation with H.V., using a two-stage process: images and overlays were inspected using a lightbox visualization, and if a problem was detected or in cases of doubt, images were subsequently inspected in a 3D orthogonal viewer. This process produced pass/fail judgements for most cases. For cases that needed further consultation, images of representative cases were discussed with H.V. until full consensus was reached; similar cases were then re-evaluated by R.M.M. with reference to those consensus decisions. For more complex cases requiring an expert neuroradiologist, F.B. was consulted, and consensus decisions were made.

### Training set and test set

To optimize parameters of the new method, a training set of 40 QC-passed cases (from 35 sites) was selected covering the full BH volume range while maximizing the number of study sites, to ensure broad applicability. The remaining QC-passed cases formed the test set used to evaluate the optimized method.

#### Pre-processing

To create the “pre-processed GdT1-weighted image” used as the input for the method, we first corrected intensity differences between odd and even slices arising from the interleaved acquisition. For this purpose, a GdT1-weighted brain mask was created by linearly registering the contrast-naive T1-weighted image to the GdT1-weighted image using FSL-FLIRT [19] with 6 degrees of freedom while removing enhancing lesions from the cost function to avoid their influence on the optimization, and applying the resulting transformation to the T1-weighted brain mask using nearest-neighbor interpolation. Then, an interslice variation correction was performed on the GdT1-weighted image as follows: if the average signal for the odd slices inside the GdT1-weighted brain mask, excluding the enhancing lesions, differed from that of the even slices by more than 2%, the signal of the odd slices was scaled such that the average became equal to that of the even slices. This step was performed to remove slice-to-slice average intensity differences. Finally, a bias field correction was performed to correct for any remaining intensity non-uniformity. This was done by running FSL-SIENAX [20] with the interslice variation corrected GdT1-weighted image and the registered brain mask minus the enhancing lesions as inputs, while passing the –B option to the FSL-FAST [21] segmentation to save the bias-corrected image. This last step was performed to remove any spatial intensity inhomogeneity remaining after the correction of slice-to-slice differences.

### Description of the proposed rule-based method

#### Brief overview

**Fig. 1:**
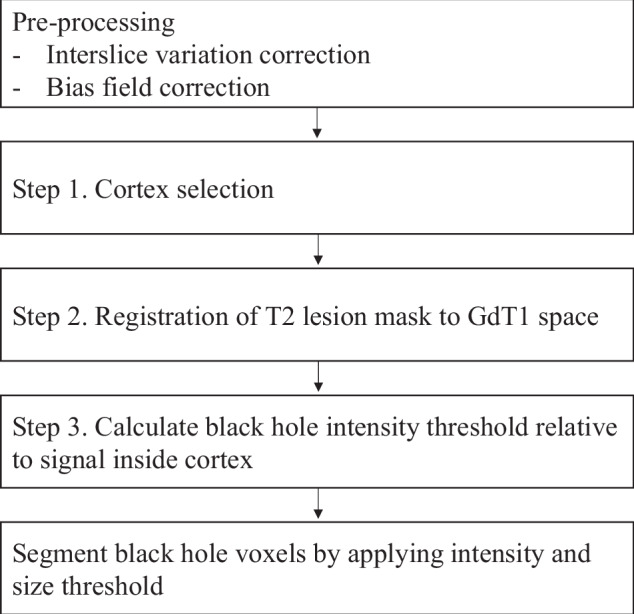
Overview of the pipeline used for the semi-automated method to segment black holes on post-gadolinium T1 (GdT1)-weighted scans. The steps correspond to the steps described in the general approach section in the “Materials and methods” section

The method follows the radiological BH definition: a T2-hyperintense lesion is considered a BH when the corresponding area on a T1-weighted image appears hypointense compared to surrounding WM and iso- to hypointense relative to cortical gray matter (GM), and measures at least 3 mm in-plane [3, 22, 23]. Because only GdT1 images with expert manual BH segmentations were available, we used post-gadolinium rather than contrast-naive T1-weighted images to develop and validate the method, similar to several previous methods [8, 10, 12, 13]. We implemented this (semi)automatically as follows: the cortex was segmented, its mean or median intensity was calculated and multiplied by a scaling factor, producing an upper intensity threshold for BHs, which was applied to the GdT1-weighted image within the co-registered PD/T2 lesion mask. Of the resulting candidate BH lesion voxels, only clusters of ≥ 3 (1 × 1 mm) voxels in-plane were retained and segmented as voxels belonging to a BH. The pipeline (Fig. 1) was implemented using FSL tools (FSL version 6.0.5.1) [24], including FSL-FLIRT [19] and FSL-SIENAX [20], as well as Python with the SciPy toolbox. The code is publicly available at https://gitlab.com/sbig/blackhole_segmentation.

### Optimization

#### General approach

To finetune the method, specific parameters of steps 1-3 in the pipeline were consecutively optimized on the training set. Starting with default options, we first optimized step 1, and then consecutively applied the best parameter in the remaining steps. Spatial overlap between semi-automatic and ground truth BH segmentations was calculated using the Dice similarity coefficient (DSC) [25], setting DSC to 1 (perfect overlap) in the special case that the ground truth and semi-automated segmentation both contained no BH voxels. For each step, the variant yielding the highest mean DSC across the training set was considered optimal.

In our dataset, differences between manual delineations on PD-weighted and GdT1-weighted images caused some voxels of ground truth BH lesion segmentations to fall outside the PD/T2 lesion mask. Because the method uses the PD/T2 lesion mask as the reference region within which to detect BHs, the ground truth BHs were masked by the manual PD/T2 lesion mask (using trilinear interpolation for the registration with no threshold). To assess its impact, we also evaluated the method’s performance without masking the ground truth.

#### Step 1. Cortex selection

Three cortex selection methods were tested, each based on FSL-SIENAX segmentations of the pre-processed GdT1-weighted image, using the GdT1-weighted brain mask (see Supplementary Material). Variants 1 and 2 were designed to handle limited WM-GM contrast, while variant 3 is GM-specific. In variants 1 and 2, the FSL-SIENAX output was masked with an MNI standard space cortex mask (Supplementary Material). To transform the MNI cortex mask to subject space, the GdT1-weighted image was registered to MNI standard space (resolution = 1 × 1 × 1 mm^3^) using FSL-FLIRT [19] with 12 degrees of freedom and applying the inverse transformation to the MNI space mask using nearest-neighbor interpolation.

##### Variant 1. Partial volume estimate WM + partial volume estimate GM masked by MNI cortex

Partial volume estimate (PVE) maps of GM and WM were summed, yielding a brain tissue PVE map, which was masked by the MNI cortex mask and converted to a cortex mask by binarization using a lower PVE threshold, varied from 0 to 1 with a step size of 0.1.

##### Variant 2. Three-class segmentation—WM + GM masked by MNI cortex

Each voxel was assigned to either GM, WM, or cerebrospinal fluid (CSF) (a “hard” segmentation); the resulting WM and GM masks were combined and masked by the MNI cortex mask.

##### Variant 3. Peripheral GM segmentation

On the cortical GM PVE maps (output: segperiph_pve1, FSL-SIENAX –r option), a threshold was applied, which varied from 0 to 1 with a step size of 0.1.

#### Step 2. Registration of the T2 lesion mask to the GdT1 space

For registration of the PD/T2 lesion mask to the GdT1-weighted space, cost function and interpolation were optimized.

##### Step 2.1. Cost function

Two cost functions were tested: correlation ratio (default) and normalized mutual information in FSL-FLIRT, with 6 degrees of freedom and otherwise default settings.

##### Step 2.2. Interpolation of the T2 lesion mask

Two interpolation methods of the PD/T2 lesion mask to the GdT1-weighted image were tested: nearest-neighbor (default); or trilinear interpolation followed by a threshold, varied from 0 to 1 with a step size of 0.1.

#### Step 3. Calculate the BH intensity threshold

The calculation of the upper intensity threshold, used to determine which voxels within PD/T2 lesion masks are candidate BH lesion voxels, was varied, taking both the cortical mean and median as starting points, and multiplying each by a scaling factor that was varied between 0.9 and 1.1 with a step size of 0.05.

### Final test

The optimized method was applied to the test set. Spatial overlap with ground truth segmentations was assessed using DSC. In addition, distances between segmentation surfaces were assessed using average symmetric surface distance (ASSD), and Hausdorff distances (full (HD), 99th percentile (HD99), and 95th percentile (HD95)), implemented using Python routines in the MedPy and SciPy libraries, and applied for each case in the test set to the full 3D segmentations of the true positive lesions. Means and standard deviations were reported for the whole test set for DSC, and for the distance metrics in the subset of cases in the test set that had both BH lesions in the ground truth segmentation and BHs according to the semi-automated method, because the calculation of distances requires two segmentations. For DSC, 95% confidence interval (CI) of the means was also calculated. For each quintile of ground truth BH volumes, mean, SD, and median DSC were calculated, and the distribution was also shown graphically. Additionally, DSC with ground truth segmentations that were not masked by the registered PD/T2 lesion mask was also calculated. Volumetric agreement with the ground truth was assessed by the intraclass correlation coefficient (ICC) for absolute agreement.

Lesion-wise performance was assessed by classifying each T2 lesion either as a BH (if it contained a cluster of BH voxels according to our method) or a non-BH lesion. Compared to ground truth, we calculated BH sensitivity = number of true positive lesions/(number of true positive lesions+number of false negative lesions), and BH specificity = number of true negative lesions/(number of true negative lesions+number of false positive lesions), both for the whole test set and the subgroups with 0–5, 6–10, 11–20, or > 20 T2 lesions.

Correlations of Expanded Disability Status Scale (EDSS) scores with both ground truth BH lesion volumes and optimized method BH lesion volumes were assessed using Spearman’s rank correlation coefficient.

IBM SPSS Statistics (version 28) was used.

## Results

Supplementary Table 3 provides QC details. Of the 323 patients (from 64 sites) included initially, three were excluded because of errors in or absence of manual BH segmentation files, leaving 320 patients. Out of these, 248 were accepted after passing lightbox image QC. This left 72 cases to be inspected using 3D orthogonal viewer QC. Out of those 72 cases, four were excluded because of severe noise, while 68 cases passed this second round of QC (Supplementary Table 3). This left a total of 248 + 68 = 316 QC-passed cases. Out of these 316 cases, 40 cases (from 35 sites) were selected as the training set. Of the remaining 276 cases, the final optimized method produced registration errors in two cases, and these were excluded, yielding a final test set of *N* = 274 from 60 sites (and a final total of 274 + 40 = 314 cases across test and training sets combined). Table 1 lists demographics.

**Table 1:** Demographics of the included patients in the training and test set

Variables	Training set (*N* = 40)	Test set (*N* = 274)
Age (years), mean (SD)	31.38 (8.68)	31.78 (8.39)
Sex (female), *N* (%)	23 (57.5)	168 (61.3)
EDSS, median (IQR)	1.5 (1.5-2.0)	1.5 (1.0–2.0)
T1 black hole lesion volume (mL), mean (SD), median (IQR)	3.10 (1.92), 2.88 (1.17–5.03)	0.69 (1.10), 0.25 (0.06–0.84)
Study sites (*N*)	35	60

Training set was optimized by ensuring variation in study sites and black hole lesion volume

*EDSS* Expanded Disability Status Scale, *IQR* interquartile range, *SD* standard deviation

### Optimization

Table 2 lists results of the optimization, showing optimal DSC results for each step; Supplementary Table 4 provides expanded results. Supplementary Table 5 provides optimization results obtained without masking ground truth BH segmentations by PD/T2 lesion masks.

**Table 2:** The best results of the different steps during the optimization process resulting in the optimal combination of parameters of the pipeline

Step 1. Cortex selection				
***Cortex selection***	**Cost function**	**Interpolation of the T2 lesion mask**	**Calculate black hole intensity threshold**	**DSC** (Mean ± SD)
*WM*_*PVE*_ + *GM*_*PVE*_ + *MNI, threshold 0.8*^*#*^	corratio	nn	mean × 1.0	0.532 ± 0.172*
*WM*_*class*_ *+ GM*_*class*_ + *MNI*	corratio	nn	mean × 1.0	0.525 ± 0.175
*Peripheral GM, threshold 1.0*^*#*^	corratio	nn	mean × 1.0	0.452 ± 0.191

Step 2.1. Cost function				
**Cortex selection**	***Cost function***	**Interpolation of the T2 lesion mask**	**Calculate black hole intensity threshold**	**DSC** (Mean ± SD)
WM_PVE_ + GM_PVE_ + MNI, threshold 0.8	*normmi*	nn	mean × 1.0	0.559 ± 0.167*
	*corratio*	nn	mean × 1.0	0.532 ± 0.172

Step 2.2. Interpolation				
**Cortex selection**	**Cost function**	***Interpolation of the T2 lesion mask***	**Calculate black hole intensity threshold**	**DSC** (Mean ± SD)
WM_PVE_ + GM_PVE_ + MNI, threshold 0.8	normmi	*trilinear threshold 0.6*^*#*^	mean × 1.0	0.560 ± 0.167*
		*nn*	mean × 1.0	0.559 ± 0.167

Step 3. Calculate black hole intensity threshold				
**Cortex selection**	**Cost function**	**Interpolation of the T2 lesion mask**	***Calculate black hole intensity threshold***	**DSC** (Mean ± SD)
WM_PVE_ + GM_PVE_ + MNI, threshold 0.8	normmi	trilinear threshold 0.6	*mean *× *1.0*^*#*^	0.560 ± 0.167*
			*median *× *1.0*^*#*^	0.556 ± 0.168

*corratio* correlation ratio, *DSC* mean Dice similarity coefficient, *GM* gray matter, *MNI* Montreal Neurological Institute standard brain, *nn* nearest-neighbor, *normmi* normalized mutual information, *SD* standard deviation, *WM* white matter, *WM*_class_; *GM*_*class*_ = WM or GM voxels from “hard” segmentation of brain tissue into three classes, *WM*_PVE_; *GM*_*PVE*_ voxelwise maps of partial volume estimates for WM and GM, respectively

The * symbol indicates the best result for a specific step. Italic font highlights the parameter that is tested in each step. ^#^Note that thresholds (steps 1 and 2.2) and scaling factors (step 3) were also varied, but only the best results are shown for the succinctness of the table; Supplementary Table 4 provides additional results

In summary, the best results were obtained by:

- Step 1. Cortex selection: sum of PVE-WM and PVE-GM masked by MNI cortex (variant 1), with a lower threshold of 0.8.
- Step 2.1. Cost function: normalized mutual information.
- Step 2.2. Interpolation of T2 lesion mask: trilinear, with a lower threshold of 0.6.
- Step 3. Calculate BH intensity threshold: mean, with multiplication factor 1.0.

### Final test

#### Volume evaluation

**Fig. 2:**
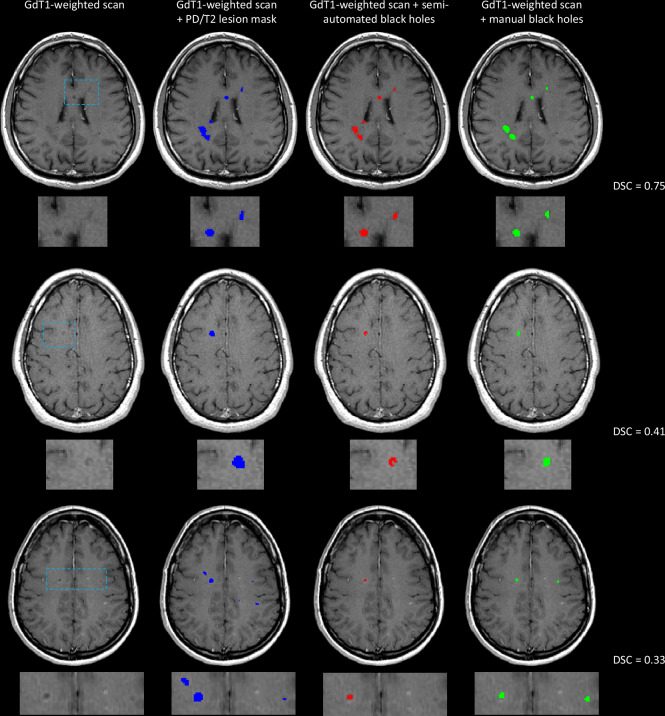
Examples of black holes segmented by the semi-automated method within the (registered) PD/T2 lesion mask on post-gadolinium T1 (GdT1)-weighted scans. The ground truth is also shown. A zoomed-in portion is shown below each scan; the blue dotted line corresponds to this part on the images. DSC, Dice similarity coefficient (*whole brain*)

**Fig. 3:**
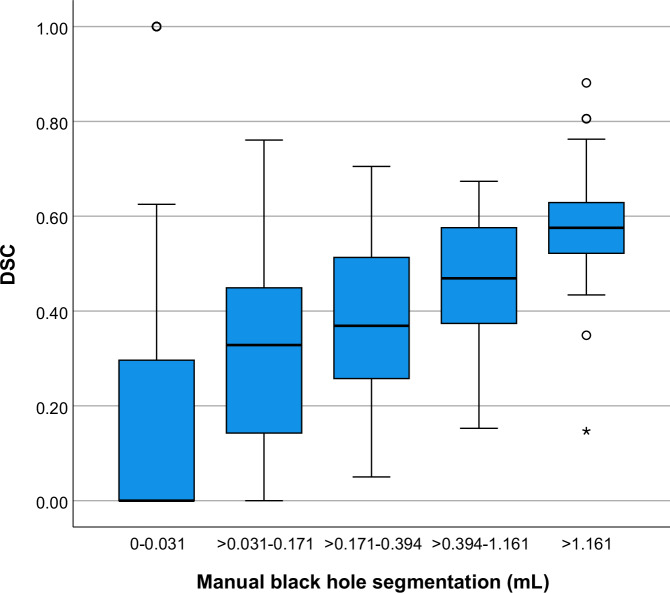
Boxplots showing the Dice similarity coefficient (DSC) for each quintile based on total ground truth black hole volume (in mL). An asterisk indicates datapoints with more than three times the interquartile range, and a circle indicates datapoints between 1 and 3 times the interquartile range

**Fig. 4:**
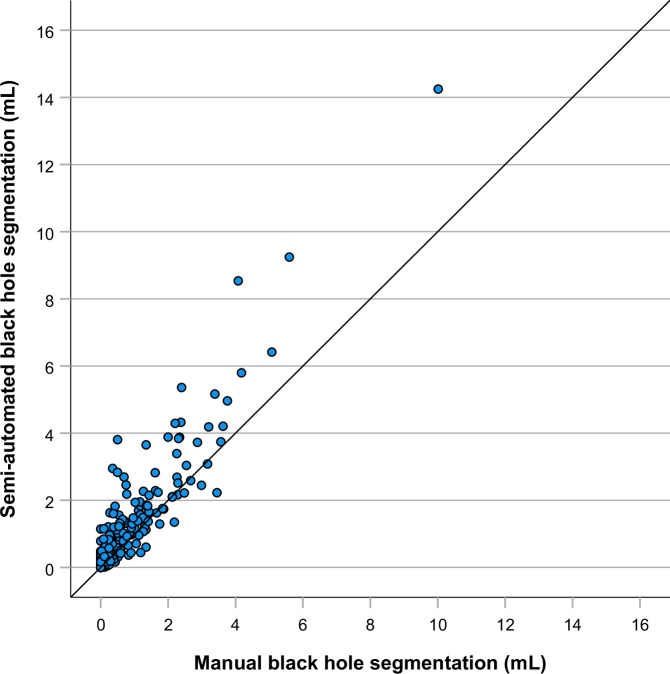
Scatterplot showing the agreement between the segmentation of black hole volumes (in mL) by the semi-automated method and the manual segmentation (ground truth; in mL). The identity line, reflecting perfect agreement, is shown for reference

Figure 2 shows examples of segmentations. The optimized method yielded a mean DSC on the test set of 0.394 ± 0.257, 95% CI [0.363, 0.425]. Not masking the ground truth with the PD/T2 lesion mask resulted in a mean DSC of 0.365 ± 0.246, 95% CI [0.336, 0.394]. Figure 3 illustrates DSC for each quintile of total ground truth BH volumes: mean ( ± SD) and median DSC steadily increased with total BH volume, from 0.218 ± 0.387 and 0.0 in the lowest quintile to 0.582 ± 0.116 and 0.576 in the highest quintile. The ICC for absolute agreement with ground truth volumes was 0.84, 95% CI [0.72, 0.90]. Figure 4 shows a scatterplot. Distance metrics were calculated on the subset of 222 patients with BH lesions in both the gold standard and semi-automated segmentations; means and standard deviations were: ASSD, 0.87 ± 0.87 mm; HD, 6.04 ± 4.42 mm; HD99, 5.17 ± 3.75 mm; HD95, 3.71 ± 2.91 mm.

#### Lesion-wise evaluation

**Fig. 5:**
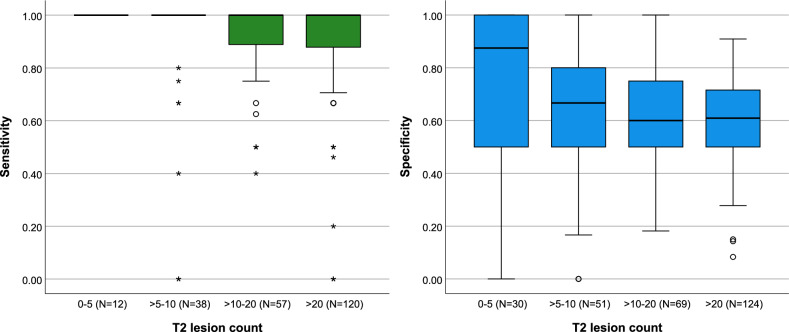
Sensitivity (left) and specificity (right) for detecting black hole lesions depicted as boxplots across different groups based on the corresponding T2 lesion count. An asterisk indicates datapoints with more than three times the interquartile range, and a circle indicates datapoints between 1 and 3 times the interquartile range

Mean BH sensitivity was 0.91 ± 0.19 (determined in *N* = 227 patients with BHs); mean specificity was 0.62 ± 0.22 (*N *= 274 patients). Figure 5 shows sensitivity and specificity in the subgroups of the test set with 0–5, 6–10, 11–20, or > 20 T2 lesions.

#### Correlation with clinical disability

Spearman’s rank correlation coefficients with EDSS scores were 0.182 (*p* = 0.003) for ground truth BH volumes, and 0.183 (*p *= 0.003) for the optimized method’s BH volumes.

## Discussion

In this study, we developed a publicly available intensity-based semi-automated method to segment “black hole” (BH) MS lesions on post-gadolinium T1 (GdT1)-weighted images within a pre-existing T2 lesion mask, following the same rules as radiologists. After optimization on a training set (*N* = 40), the method showed acceptable overlap with manual reference segmentations (mean DSC = 0.394) in a multi-center test set (*N* = 274). The main advances of the proposed method are that it is robust to multi-center acquisition variability; that it adheres to the radiological rules for defining BH lesions; that it was validated in a large multi-center dataset; and that its code is publicly available.

BH lesions mostly represent neuronal tissue destruction and axonal loss [5]. BH lesion counts and volumes exhibited stronger clinical correlations than T2 lesions [4] and serve as secondary endpoints in clinical treatment trials in MS [6, 7]. Therefore, the assessment of MS disease burden from standard MS protocol images could be improved through reliable BH lesion quantification. Our method exhibited high sensitivity (mean 0.91), missing only few BHs, independent of T2 lesion count, which suggests this method could facilitate BH lesion detection in a clinical setting, complementing information on T2 and contrast-enhancing lesions. However, specificity was lower (mean 0.62), mainly due to false positives, although specificity increased with T2 lesion count. Potential technical approaches to reducing the number of false positives should be explored, including incorporating anatomical, lesion-wise, and/or slice-specific adjustments that may include AI approaches. However, until such improvements have been validated, clinical use of this method will require correction of false-positive BH lesions by a neuroradiological expert. The method’s high lesion sensitivity comes at the cost of relatively low specificity, limiting its use as a fully automated biomarker. Confirming their clinical relevance, BH volumes were weakly but statistically significantly correlated with EDSS scores despite the limited EDSS variability in this cohort, with nearly identical correlation coefficients for the new method and the ground truth.

This method can handle different scanners and acquisitions, as in clinical practice and multi-center trials, which was achieved by optimizing on such data. Variations across sites or images of WM, GM, and CSF intensities are handled by using the FSL-FAST segmentation algorithm [21] implemented in FSL-SIENAX [20] to find the tissue classes. Our method allows analysis of legacy data to extract BH volumes and counts, but also provides a starting point for extracting, e.g., shape and intensity features. Such features could provide new insights into MS disease processes or its distinguishing characteristics through radiomics studies such as [26–30].

The optimal BH-defining intensity threshold derives from a combination of GM, WM, and mixed WM-GM voxels in the cortical region, rather than pure cortical GM voxels. This perhaps counterintuitive optimization result probably reflects limited WM-GM tissue contrast in many 2D T1-weighted images, which yields inaccurate GM segmentations. By overcoming this hurdle, the optimization has yielded a widely applicable BH segmentation method that showed acceptable performance for both spatial and volumetric agreement in 274 cases from 60 sites. Nonetheless, the achieved DSC within a multi-center context is lower than previously published direct comparisons of other BH segmentation methods with manual segmentations. However, those studies analyzed only single-center data from smaller groups (Supplementary Table 1) [9–12]. Several factors could contribute to this discrepancy, such as dataset heterogeneity owing to the use of multiple scanners versus a single scanner. Lesion size distributions could also differ between our study and other work, especially because patients in our sample were included in the trial at the very first onset of MS. Therefore, as expected, they have very low BH lesion volumes compared to people with MS with longer disease duration. Lastly, there are inherent constraints to the thresholding approach employed in our method. A single global threshold may not provide the best results across a full 3D image volume. It is conceivable that an anatomically varying threshold might be more accurate. Although we optimized the parameters of our method by maximizing mean DSC, which penalizes both false positive and false negative voxels, it is possible that the single global threshold resulting from that optimization can be too lenient. The high sensitivity and low specificity at the lesion level are consistent with a threshold that is too lenient, which is a known characteristic of liberal thresholding methods. To better interpret our DSC values in a multi-center context, we also compared to multi-center T2 lesion segmentation performance, which exhibited a mean DSC from 0.26 to 0.44 across multiple methods as reviewed in ref. [31], comparable to our mean DSC of 0.394. Although our method showed some over-segmentation, the absolute volumetric agreement with the ground truth was good (ICC = 0.84).

In the training set, DSC with ground truth was higher (mean DSC = 0.560) than in the test set, comparable to previously described BH segmentation methods in single centers (mean DSC ranging from 0.53–0.73 in 10–40 subjects [9–12] (Supplementary Table 1). The training set was enriched for larger lesion volumes to ensure broad applicability of the method, causing somewhat worse performance in the remaining test set because these early MS patients mostly had small BH lesion volumes. Within the test set, DSC was also lowest for small BH lesion volumes (Fig. 3). Further optimization towards cases with low BH volumes should be investigated in future work, although smaller lesion volumes do typically yield poorer overlap through relatively harsh penalization of small errors, as reported for lesion segmentation in MS and elsewhere [14, 32–35]. It is important to stress that, because of this effect, the specific value of DSC obtained in a given dataset depends not only on the quality of the segmentation but also on the size (expressed in voxels) of the objects under investigation. Those objects vary from study to study, and for this reason alone, it is difficult to compare DSC values between studies. The present study included only patients who were very early in the disease course and, therefore, had very low BH lesion loads compared to the typical MS patient. Additionally, in the case of small objects, DSC is very sensitive to small segmentation errors. In the context of optimizing a segmentation method, that sensitivity is a strength of DSC rather than a weakness. Since the aim of the optimization is to minimize even such small errors, the fact that they are severely penalized by DSC is a useful attribute of the metric. This sensitivity, therefore, makes DSC and similar overlap-based measures highly suitable for evaluating lesion segmentations.

To provide a more comprehensive assessment, we also reported metrics based on distances between segmentations. We specifically chose to calculate the distance metrics only for the true positive lesions in order to avoid the situation where those distance metrics are completely determined by the distance between a false negative or false positive lesion and the nearest lesion in the other segmentation, which would yield a rather arbitrary number that is unrelated to segmentation performance. The ASSD showed that the average distance was smaller than the in-plane voxel size, suggesting reasonable accuracy of our semi-automated segmentation. Second, we reported three variants of the Hausdorff distance: for the full HD, the mean was 6.04 mm, and there was quite some variability across patients (SD 4.43 mm), which is understandable given that HD is determined by the single largest distance that occurs. The 99th and 95th percentile Hausdorff distances HD99 and HD95 have the benefit of removing such extreme outliers, and in our study yielded mean values of 5.17 and 3.71 mm, respectively. It should be noted that there were many cases with small overall BH lesion volumes: out of the 222 cases in the distance analysis, 19 had total ground truth BH volumes smaller than 20 voxels, and 95 had total ground truth BH volumes smaller than 100 voxels. This implies that the percentile distance metrics have to be interpreted with caution. Distance measures can offer important additional information for large structures, especially in cases where a segmentation error has a small relative volume but extends relatively far from the ground truth segmentations. However, the added value here is limited because BH lesions are small and voxels outside T2 lesions were excluded.

To maximize the method’s generalizability, we included 35 centers in the training set, thereby avoiding optimization towards one particular scanner or acquisition protocol. Although most centers were represented by only one scan in the training set, which could have biased the optimization, the method is robust (Supplementary Table 4): minor parameter changes resulted in minor performance loss. Acquisition parameters for the 2D T1-weighted images used here fell within pre-defined, typical ranges, but with deviating acquisition parameters, re-optimization may be needed. That limits the clinical applicability of the method in its present form. More generally, since we optimized our method on trial data with pre-defined acquisition criteria, its performance in real-world clinical data may be poorer. We prioritized generalizability of the method, but scanner and acquisition-specific re-optimization could further improve performance, e.g., in images with good WM-GM contrast, re-optimization may yield a purely GM-based BH intensity threshold, as in our variant 3.

Deep learning methods are increasingly becoming the standard approach in image segmentation problems, and in future work, we aim to address the problem of BH MS lesion segmentation using such techniques as well. Applying transfer learning to tune more general deep learning segmentation models to the problem of BH MS lesion segmentation might overcome the limitation that datasets with voxel-wise labels of BH lesions are scarce. Potentially, the use of foundation models could provide an even more generic starting point for such repurposing toward the BH lesion segmentation problem, while task synthesis might enable leveraging the advantages of deep learning for optimized performance on subtasks and their combination. In the current method, we chose not to incorporate deep learning methods to stay close to the radiological definition, minimize pre-processing, provide interpretable and explainable results, avoid the necessity of expensive computing resources, and minimize the ecological footprint of the method. Nonetheless, we intend to explore the possibilities of deep learning for this particular problem; the current method could be used to provide training data for future deep learning methods, which could hopefully be trained to become even more robust to differences between scanners and acquisition protocols than the current method.

## Limitations

The most important limitation of the method is that it segments BH lesions on post-gadolinium rather than contrast-naive T1-weighted images. This choice was made because we only had access to manual segmentations of BH lesions that were made on post-gadolinium images. Although BH lesions are originally defined on contrast-naive T1-weighted images, defining them on GdT1-weighted images is not unprecedented [22]; BH lesion volumes obtained from contrast-naive and GdT1-weighted images are highly intercorrelated [36]; and some investigators have explicitly chosen to use GdT1-weighted images for BH quantification in order to exclude active lesions and focus on persistent BH lesions [13]. Interestingly, 4 out of the 7 previous methods papers cited in Supplementary Table 1 also used post-gadolinium rather than contrast-naive T1-weighted images [8, 10, 12, 13]. In our method specifically, the use of GdT1 images could negatively impact the result in several ways. Despite the brain and contrast-enhancing lesion masking employed, some high-intensity regions, such as veins, could still affect image analysis, including registration and tissue intensity distribution fitting. Moreover, the presence of small amounts of contrast agent locally, whether in lesions, veins, or possibly in non-lesional tissue, would cause intensity changes that could hamper image analysis by changing signal intensity distributions, altering the WM-GM tissue contrast, or by obscuring BH lesions [22]. BH lesions that are isointense to WM due to subtle leakage of contrast agent would be missed by both the human expert rater and our method. The validation against manual segmentations showed acceptable performance, indicating that the method can cope with the technical limitations to a reasonable extent. Nonetheless, the clinical applicability of the currently proposed method is diminished by the reliance on post-gadolinium images, especially considering recent efforts to limit the use of gadolinium-based MR contrast agents in MS. The method requires re-optimization and validation before it can be reliably applied to contrast-naive T1-weighted images. Unfortunately, we were unable to include that in the present study due to a lack of available manually segmented data.

A limitation of the current validation study is that no fully independent validation was performed because no additional dataset with expert manual BH lesion segmentations was available. This lack of independent validation on external data presents a limitation to the broader application of the method.

Another limitation of the method is that it is recommended to be used with human intervention. Especially when detection at the lesion level is important, as in the clinical setting, the method produces too many false positives to be used on its own. Instead, this method could be used as a tool to aid and speed up the process of the radiologist when identifying BHs. This way, the semi-automatically segmented BH lesions could still be checked by the rater, and false positives could be removed.

While our method fits the definition of BH lesions on 2D T1-weighted images, recent MS brain imaging recommendations favor 3D acquisition [37]. Future work should encompass 3D T1-weighted acquisitions, accommodating their typically stronger T1-weighting and its effects on BH lesion classification [12, 38].

Small inconsistencies between manual T2 and BH lesion segmentations, made on different images, sometimes caused a BH to partially fall outside the co-registered mask of the corresponding T2 lesion, which violates the BH definition and was treated as a (minor) inaccuracy of the manual segmentations: ground truth BH segmentations were masked by their respective co-registered T2 lesion masks to ensure consistency. The effect was small: without masking the ground truth, DSC in the test set was 0.365 ± 0.246, compared to 0.394 ± 0.257 with masking. Unmasked ground truth BH segmentations also yielded the same optimization result in all steps except cortex selection (Supplementary Table 5). For cortex selection, an extreme 1.0 threshold gave slightly higher mean DSC than 0.8 (0.500579 vs. 0.500441), but because the difference was so small and the intermediate threshold of 0.9 gave poorer mean DSC than either 0.8 or 1.0, we considered the 0.8 threshold to constitute the more stable optimum than the extreme value of 1.0.

Our semi-automated method’s reliance on existing T2 lesion masks is a limitation, and fully automated methods might seem preferable. However, only two published fully automated methods can function with the image types typically available in legacy data (2D T1-weighted, PD-weighted, and T2-weighted) [8, 14], and their performances are unclear: one did not report overlap with manual segmentations [8], while the other reported DSC for their 10 patients only visually in a graph [14]. The other two fully automated methods [9, 11] require different image types and only one measured overlap with manual segmentations, reporting DSC of 0.53 ± 0.14 from single-center cross-validation in 40 subjects [11], which is comparable to the optimized performance of our method in our 40-patient multi-center training set (DSC of 0.56 ± 0.17) (Supplementary Table 1). The fact that our method is publicly available, works on legacy data image types, and performs adequately in multi-center data, in our view, outweighs the limitation of relying on external T2 lesion segmentation. Although ruling out the site-related variation introduced by automatic tools [39], the manual T2 lesion segmentation used here is labor-intensive and could be replaced by automated methods [39, 40], to be validated in future work, with particular attention to using FLAIR images (as currently recommended clinically) [37] to segment T2 lesions, especially high-resolution isotropic 3D-FLAIR. Such work should assess the possibly higher risk of false positive BHs due to a possible increase in the number of MS lesions detected [41, 42], and investigate if FLAIR lesion masks with superior spatial resolution yield more accurate BH lesion segmentations. While the proposed method does not present a primary novel algorithmic advancement, its validation against ground truth manual segmentations in a large-scale, heterogeneous multi-center dataset constitutes a substantial empirical contribution to the field that provides confidence in the performance of the method.

In conclusion, our publicly available, semi-automatic BH lesion segmentation method shows acceptable performance in multi-center data and can help to obtain quantitative information on BH lesions when T2 lesion segmentations are available. The method is recommended to be used as a tool to aid the radiologist, but it is not recommended to be used entirely without human intervention.

## Supplementary information

Supplementary information

## Abbreviations

BH: Black hole
CI: Confidence interval
CSF: Cerebrospinal fluid
DSC: Dice similarity coefficient
EDSS: Expanded Disability Status Scale
FLAIR: Fluid-attenuated inversion recovery
FSL: FMRIB software library
GdT1: Post-gadolinium T1
GM: Gray matter
ICC: Intraclass correlation coefficient
MS: Multiple sclerosis
PD: Proton-density
PVE: Partial volume estimate
QC: Quality control
WM: White matter

## References

[1] Lassmann H, Brück W, Lucchinetti CF (2007) The immunopathology of multiple sclerosis: an overview. Brain Pathol 17:210–218 17388952 10.1111/j.1750-3639.2007.00064.x PMC8095582

[2] Popescu BF, Lucchinetti CF (2012) Pathology of demyelinating diseases. Annu Rev Pathol 7:185–217 22313379 10.1146/annurev-pathol-011811-132443

[3] van Walderveen MA, Barkhof F, Hommes OR et al (1995) Correlating MRI and clinical disease activity in multiple sclerosis: relevance of hypointense lesions on short-TR/short-TE (T1-weighted) spin-echo images. Neurology 45:1684–1690 7675227 10.1212/wnl.45.9.1684

[4] Zivadinov R, Leist TP (2005) Clinical-magnetic resonance imaging correlations in multiple sclerosis. J Neuroimaging 15:10S–21S 16385015 10.1177/1051228405283291

[5] Barkhof F, van Walderveen M (1999) Characterization of tissue damage in multiple sclerosis by nuclear magnetic resonance. Philos Trans R Soc Lond B Biol Sci 354:1675–1686 10603619 10.1098/rstb.1999.0511 PMC1692677

[6] Foley JF, Defer G, Ryerson LZ et al (2022) Comparison of switching to 6-week dosing of natalizumab versus continuing with 4-week dosing in patients with relapsing-remitting multiple sclerosis (NOVA): a randomised, controlled, open-label, phase 3b trial. Lancet Neurol 21:608–619 35483387 10.1016/S1474-4422(22)00143-0

[7] Hartung HP, Derfuss T, Cree BA et al (2022) Efficacy and safety of temelimab in multiple sclerosis: Results of a randomized phase 2b and extension study. Mult Scler 28:429–440 34240656 10.1177/13524585211024997

[8] Wu Y, Warfield SK, Tan IL et al (2006) Automated segmentation of multiple sclerosis lesion subtypes with multichannel MRI. Neuroimage 32:1205–1215 16797188 10.1016/j.neuroimage.2006.04.211

[9] Spies L, Tewes A, Suppa P et al (2013) Fully automatic detection of deep white matter T1 hypointense lesions in multiple sclerosis. Phys Med Biol 58:8323–8337 24216694 10.1088/0031-9155/58/23/8323

[10] Datta S, Sajja BR, He R, Wolinsky JS, Gupta RK, Narayana PA (2006) Segmentation and quantification of black holes in multiple sclerosis. Neuroimage 29:467–474 16126416 10.1016/j.neuroimage.2005.07.042 PMC1808226

[11] Valcarcel AM, Linn KA, Khalid F et al (2018) A dual modeling approach to automatic segmentation of cerebral T2 hyperintensities and T1 black holes in multiple sclerosis. Neuroimage Clin 20:1211–1221 30391859 10.1016/j.nicl.2018.10.013 PMC6224321

[12] Tam RC, Traboulsee A, Riddehough A, Sheikhzadeh F, Li DK (2011) The impact of intensity variations in T1-hypointense lesions on clinical correlations in multiple sclerosis. Mult Scler 17:949–957 21502309 10.1177/1352458511402113

[13] Tam RC, Traboulsee A, Riddehough A, Li DK (2012) Improving the clinical correlation of multiple sclerosis black hole volume change by paired-scan analysis. Neuroimage Clin 1:29–36 24179734 10.1016/j.nicl.2012.08.004 PMC3757731

[14] Harmouche R, Subbanna NK, Collins DL, Arnold DL, Arbel T (2015) Probabilistic multiple sclerosis lesion classification based on modeling regional intensity variability and local neighborhood information. IEEE Trans Biomed Eng 62:1281–1292 25546852 10.1109/TBME.2014.2385635

[15] Comi G, De Stefano N, Freedman MS et al (2017) Subcutaneous interferon beta-1a in the treatment of clinically isolated syndromes: 3-year and 5-year results of the phase III dosing frequency-blind multicentre REFLEXION study. J Neurol Neurosurg Psychiatry 88:285–294 28039317 10.1136/jnnp-2016-314843

[16] Smith SM (2002) Fast robust automated brain extraction. Hum Brain Mapp 17:143–155 12391568 10.1002/hbm.10062 PMC6871816

[17] Smith SM, Jenkinson M, Woolrich MW et al (2004) Advances in functional and structural MR image analysis and implementation as FSL. Neuroimage 23:S208–S219 15501092 10.1016/j.neuroimage.2004.07.051

[18] Mattiesing RM, Gentile G, Brouwer I et al (2022) The spatio-temporal relationship between white matter lesion volume changes and brain atrophy in clinically isolated syndrome and early multiple sclerosis. Neuroimage Clin 36:103220 36274376 10.1016/j.nicl.2022.103220 PMC9668617

[19] Jenkinson M, Bannister P, Brady M, Smith S (2002) Improved optimization for the robust and accurate linear registration and motion correction of brain images. Neuroimage 17:825–841 12377157 10.1016/s1053-8119(02)91132-8

[20] Smith SM, Zhang Y, Jenkinson M et al (2002) Accurate, robust, and automated longitudinal and cross-sectional brain change analysis. Neuroimage 17:479–489 12482100 10.1006/nimg.2002.1040

[21] Zhang Y, Brady M, Smith S (2001) Segmentation of brain MR images through a hidden Markov random field model and the expectation-maximization algorithm. IEEE Trans Med Imaging 20:45–57 11293691 10.1109/42.906424

[22] Sahraian MA, Radue EW, Haller S, Kappos L (2010) Black holes in multiple sclerosis: definition, evolution, and clinical correlations. Acta Neurol Scand 122:1–8 20003089 10.1111/j.1600-0404.2009.01221.x

[23] Nagtegaal GJ, Pohl C, Wattjes MP et al (2014) Interferon beta-1b reduces black holes in a randomised trial of clinically isolated syndrome. Mult Scler 20:234–242 23842212 10.1177/1352458513494491

[24] Jenkinson M, Beckmann CF, Behrens TE, Woolrich MW, Smith SM (2012) FSL. Neuroimage 62:782–790 21979382 10.1016/j.neuroimage.2011.09.015

[25] Dice LR (1945) Measures of the amount of ecologic association between species. Ecology 26:297–302

[26] Liu Y, Dong D, Zhang L et al (2019) Radiomics in multiple sclerosis and neuromyelitis optica spectrum disorder. Eur Radiol 29:4670–4677 30770971 10.1007/s00330-019-06026-w

[27] Luo X, Li H, Xia W et al (2024) Joint radiomics and spatial distribution model for MRI-based discrimination of multiple sclerosis, neuromyelitis optica spectrum disorder, and myelin-oligodendrocyte-glycoprotein-IgG-associated disorder. Eur Radiol 34:4364–4375 38127076 10.1007/s00330-023-10529-y

[28] Luo X, Piao S, Li H et al (2022) Multi-lesion radiomics model for discrimination of relapsing-remitting multiple sclerosis and neuropsychiatric systemic lupus erythematosus. Eur Radiol 32:5700–5710 35243524 10.1007/s00330-022-08653-2

[29] Peng Y, Zheng Y, Tan Z et al (2021) Prediction of unenhanced lesion evolution in multiple sclerosis using radiomics-based models: a machine learning approach. Mult Scler Relat Disord 53:102989 34052741 10.1016/j.msard.2021.102989

[30] Pontillo G, Tommasin S, Cuocolo R et al (2021) A combined radiomics and machine learning approach to overcome the clinicoradiologic paradox in multiple sclerosis. AJNR Am J Neuroradiol 42:1927–1933 34531195 10.3174/ajnr.A7274 PMC8583276

[31] de Sitter A, Steenwijk MD, Ruet A et al (2017) Performance of five research-domain automated WM lesion segmentation methods in a multi-center MS study. Neuroimage 163:106–114 28899746 10.1016/j.neuroimage.2017.09.011

[32] Anbeek P, Vincken KL, van Osch MJ, Bisschops RH, van der Grond J (2004) Probabilistic segmentation of white matter lesions in MR imaging. Neuroimage 21:1037–1044 15006671 10.1016/j.neuroimage.2003.10.012

[33] Sajja BR, Datta S, He R et al (2006) Unified approach for multiple sclerosis lesion segmentation on brain MRI. Ann Biomed Eng 34:142–151 16525763 10.1007/s10439-005-9009-0 PMC1463248

[34] Schmidt P, Gaser C, Arsic M et al (2012) An automated tool for detection of FLAIR-hyperintense white-matter lesions in Multiple Sclerosis. Neuroimage 59:3774–3783 22119648 10.1016/j.neuroimage.2011.11.032

[35] Steenwijk MD, Pouwels PJW, Daams M et al (2013) Accurate white matter lesion segmentation by k nearest neighbor classification with tissue type priors (kNN-TTPs). Neuroimage Clin 3:462–469 24273728 10.1016/j.nicl.2013.10.003 PMC3830067

[36] O’Riordan JI, Gawne Cain M, Coles A et al (1998) T1 hypointense lesion load in secondary progressive multiple sclerosis: a comparison of pre versus post contrast loads and of manual versus semi automated threshold techniques for lesion segmentation. Mult Scler 4:408–412 9839300 10.1177/135245859800400502

[37] Wattjes MP, Ciccarelli O, Reich DS et al (2021) 2021 MAGNIMS-CMSC-NAIMS consensus recommendations on the use of MRI in patients with multiple sclerosis. Lancet Neurol 20:653–670 34139157 10.1016/S1474-4422(21)00095-8

[38] Kocsis K, Szabo N, Toth E et al (2021) Two Classes of T1 Hypointense Lesions in Multiple Sclerosis With Different Clinical Relevance. Front Neurol 12:619135 33746876 10.3389/fneur.2021.619135 PMC7966518

[39] Lladó X, Oliver A, Cabezas M et al (2012) Segmentation of multiple sclerosis lesions in brain MRI: a review of automated approaches. Inf Sci 186:164–185

[40] García-Lorenzo D, Francis S, Narayanan S, Arnold DL, Collins DL (2013) Review of automatic segmentation methods of multiple sclerosis white matter lesions on conventional magnetic resonance imaging. Med Image Anal 17:1–18 23084503 10.1016/j.media.2012.09.004

[41] Moraal B, Roosendaal SD, Pouwels PJ et al (2008) Multi-contrast, isotropic, single-slab 3D MR imaging in multiple sclerosis. Eur Radiol 18:2311–2320 18509658 10.1007/s00330-008-1009-7

[42] Wattjes MP, Lutterbey GG, Harzheim M et al (2006) Imaging of inflammatory lesions at 3.0 Tesla in patients with clinically isolated syndromes suggestive of multiple sclerosis: a comparison of fluid-attenuated inversion recovery with T2 turbo spin-echo. Eur Radiol 16:1494–1500 16550354 10.1007/s00330-005-0082-4

