# Supplementary material for: Rule-based semi-automated method to segment black hole multiple sclerosis lesions on post-gadolinium 2D T1-weighted brain images
Source: Eur Radiol. 2026 May 2;36(9):7242–55. doi: 10.1007/s00330-026-12577-6 (PMC13451240; doi:10.1007/s00330-026-12577-6)
Supplement: Supplementary file 1 [file 330_2026_12577_MOESM1_ESM.pdf]

Rule-based semi-automated method to segment black hole multiple sclerosis lesions on post-gadolinium 2D T1-weighted brain images

ELECTRONIC SUPPLEMENTARY MATERIAL

Supplementary Methods

MNI space cortex mask creation

The MNI standard space cortex mask used in variants 1 and 2 of the cortex selection step was created by binarizing ${FSLDIR}/data/atlases/HarvardOxford/HarvardOxford-cort-maxprob-thr25-1mm from the Harvard-Oxford atlas distributed with FSL (https://fsl.fmrib.ox.ac.uk/fsl/fslwiki/Atlases).

Supplementary Tables

Supplementary Table 1. Characteristics of previously published black hole lesion segmentation method validation compared to the current work.

Paper	Required images	Fully automated?	Code available?	Number of patients	Single/multi-center	Comparison with manual segmentation?	Reported DSC with manual for best method variant	Reported ICC with manual for best method variant
Datta et al., 2006 [10]	FLAIR, PD, T2, T1, GdT1	No (Requires operator to remove extrameningeal tissues)	No	14	Single	Yes	0.73±0.11	-
Wu et al., 2006 [8]	PD, T2, GdT1	Yes	No	6	Single	Yes	-	0.95
Tam et al., 2011 [12]	T2, GdT1	No (Requires lesion identification by radiologist)	No	24	Single	Yes	Mean 0.69 (no SD reported)	-
Tam et al., 2012 [13]	T2, GdT1	No (Requires lesion identification by radiologist)	No	247	Multi (14 sites)	No	-	-
Spies et al., 2013 [9]	MPRAGE (no Gd)	Yes	No	10	Single	Not for real patient data	-	-
Harmouche et al., 2015 [14]	PD, T2, T1 (no information about Gd)	Yes	No	10	Single	Yes	Only reported visually in graph; individual patient DSC scores ranging from ~0.26 to ~0.68	-
Valcarcel et al., 2018 [11]	3D-FLAIR, 3D-T2, 3D-T1 (no Gd)	Yes	Yes	40 (using cross-validation)	Single	Yes	0.53±0.14	-
Current manuscript	*GdT1, T2 lesion masks*	*No (Requires T2 lesion masks)*	*Yes*	*274 (independent test set)*	*Multi (60 sites)*	*Yes*	*0.39±0.26*	*0.85 (ICC for absolute agreement)*

DSC = Dice similarity coefficient, FLAIR = fluid-attenuated inversion recovery, Gd = gadolinium, GdT1 = post-gadolinium T1, ICC = intraclass correlation coefficient, MPRAGE = magnetization prepared rapid gradient echo, PD = proton-density, SD = standard deviation. Note that the reference list can be found in the main manuscript.

Supplementary Table 2. Image acquisition parameters.

Acquisition parameter	T1-weighted (both with and without contrast agent)	Dual-echo (PD-/T2-weighted images)
**Sequence**	2D spin-echo	2D dual-echo spin-echo
**Plane**	Axial	Axial
**TR (ms)**	400-600	2000-3000
**TE1/TE2 (ms)**	10-16	20-30/80-100
**Number of slices**	46	46
**Slice Thickness (mm)**	3	3

mm = millimeter, ms = milliseconds, PD = proton-density, TE1/TE2 = echo time 1/2, TR = repetition time.

Supplementary Table 3. Input image quality control.

**Image quality aspect****	**Lightbox inspection (1st round)**<br><br>(N=320 cases, of which 248 accepted at this stage)		**3D orthogonal viewer inspection (2nd round)**<br><br>(N=72 cases subjected to further inspection)		**Exclusions**
Artefact severity	Mild	Severe	Mild	Severe	
Zipper artefact	0	-	2	-	-
Ghosting artefact	1	-	10	-	-
Ringing artefact	8	-	1	-	-
Suboptimal image contrast	5	-	13	-	-
Image inhomogeneity	1	-	13	-	-
Noise	17	-	35	4	4

**Note that some cases have multiple artefacts.

Supplementary Table 4. Expanded results of optimization.

Results of the optimization process for different parameter settings to allow assessment of sensitivity of the performance to small parameter changes. Within each variant, in case of multiple possibilities, e.g., different threshold values, the optimal result is highlighted by right adjustment. Within each step of the segmentation method, the overall optimal result is additionally marked in bold font and followed by an asterisk.

**Step 1. Cortex selection**				
Cortex selection	**Cost function**	**Interpolation of the T2 lesion mask**	**Calculate black hole intensity threshold**	**DSC±SD**
WM_{PVE} + GM_{PVE} + MNI,<br>*threshold 0.6*<br>*threshold 0.7*<br>***threshold 0.8***<br>*threshold 0.9*<br>*threshold 1.0*	corratio	nn	mean×1.0	 <br>0.528735±0.174<br>0.531424±0.173<br>**0.532424±0.172***<br>0.531878±0.172<br>0.532222±0.172
WM_{class} + GM_{class} + MNI	corratio	nn	mean×1.0	0.525118±0.175
Peripheral GM,<br>*threshold 0.7*<br>*threshold 0.8*<br>*threshold 0.9*<br>*threshold 1.0*	corratio	nn	mean×1.0	 0.433232±0.192<br>0.447053±0.192<br>0.451659±0.191<br>0.452467±0.191
**Step 2.1. Cost function**				
**Cortex selection**	***Cost function***	**Interpolation of the T2 lesion mask**	**Calculate black hole intensity threshold**	**DSC±SD**
WM_{PVE} + GM_{PVE} + MNI, threshold 0.8	***normmi***	nn	mean×1.0	**0.558592±0.167***
	corratio	nn	mean×1.0	0.532424±0.172
**Step 2.2. Interpolation**				
**Cortex selection**	**Cost function**	***Interpolation of the T2 lesion mask***	**Calculate black hole intensity threshold**	**DSC±SD**
WM_{PVE} + GM_{PVE} + MNI,<br>threshold 0.8	normmi	*trilinear,*<br>*threshold 0.0*<br>*threshold 0.1*<br>*threshold 0.2*<br>*threshold 0.3*<br>*threshold 0.4*<br>*threshold 0.5*<br>***threshold 0.6***<br>*threshold 0.7*<br>*threshold 0.8*	mean×1.0	 0.452361±0.159<br>0.517616±0.172<br>0.530770±0.174<br>0.535320±0.174<br>0.536372±0.174<br>0.536434±0.174<br>**0.559948±0.167***<br>0.558083±0.166<br>0.553165±0.163

		threshold 0.9<br>*threshold 1.0*		0.517572±0.170<br>0.337320±0.169
		nn	mean×1.0	0.558592±0.167
**Step 3. Calculate black hole intensity threshold**				
**Cortex selection**	**Cost function**	**Interpolation of the T2 lesion mask**	***Calculate black hole intensity threshold***	**DSC±SD**
WM_{PVE} + GM_{PVE} + MNI, threshold 0.8	normmi	trilinear threshold 0.6	*mean×0.90*<br>*mean×0.95*<br>***mean×1.0***<br>*mean×1.05*<br>*mean×1.10*	0.433275±0.210<br>0.522705±0.184<br>**0.559948±0.167***<br>0.513434±0.171<br>0.467346±0.167
			median×0.90<br>*median×0.95*<br>*median×1.0*<br>*median×1.05*<br>*median×1.10*	0.434399±0.212<br>0.519072±0.187<br>0.555935±0.168<br>0.510239±0.171<br>0.467060±0.167

corratio = correlation ratio, DSC = mean Dice similarity coefficient, GM = gray matter, MNI = Montreal Neurological Institute standard brain, nn = nearest-neighbor, normmi = normalized mutual information, SD = standard deviation, WM = white matter, WM_{class}; GM_{class} = WM or GM voxels from "hard" segmentation of brain tissue into three classes, WM_{PVE}; GM_{PVE} = voxelwise maps of partial volume estimates for WM and GM, respectively. The * symbol indicates the best result for a specific step. Italic font highlights the parameter that is tested in each step. Note that mean DSC values are provided in this Supplementary Table to six decimals in order to allow the reader to appreciate small performance differences between different parameter settings.

Supplementary Table 5. Optimization results without masking the ground truth.

Results of evaluations in training set following optimization scheme as in main paper, but this time <u>without</u> masking the ground truth black hole lesion masks with the corresponding PD/T2 lesion masks (i.e., allowing occasional minor mismatches). Within each variant, in case of multiple possibilities, e.g., different threshold values, the optimal result is highlighted by right adjustment. Within each step of the segmentation method, the overall optimal result is additionally marked in bold font and followed by an asterisk.

**Step 1. Cortex selection**				
Cortex selection	**Cost function**	**Interpolation of the T2 lesion mask**	**Calculate black hole intensity threshold**	**DSC±SD**
WM_{PVE} + GM_{PVE} + MNI,<br>*threshold 0.7*<br>***threshold 0.8***<br>*threshold 0.9*<br>*threshold 1.0*	corratio	nn	mean×1.0	 0.498789±0.166<br>**0.500441±0.164***<br>0.500159±0.165<br>0.500579±0.164
WM_{class} + GM_{class} + MNI	corratio	nn	mean×1.0	0.490987±0.167
Peripheral GM,<br>*threshold 0.7*<br>*threshold 0.8*<br>*threshold 0.9*<br>*threshold 1.0*	corratio	nn	mean×1.0	 0.399651±0.182<br>0.413091±0.182<br>0.417691±0.183<br>0.418481±0.183
**Step 2.1. Cost function**				
**Cortex selection**	***Cost function***	**Interpolation of the T2 lesion mask**	**Calculate black hole intensity threshold**	**DSC±SD**
WM_{PVE} + GM_{PVE} + MNI,<br>threshold 0.8	***normmi***	nn	mean×1.0	**0.526951±0.164***
	corratio	nn	mean×1.0	0.500441±0.164
**Step 2.2. Interpolation**				
**Cortex selection**	**Cost function**	***Interpolation of the T2 lesion mask***	**Calculate black hole intensity threshold**	**DSC±SD**
WM_{PVE} + GM_{PVE} + MNI,<br>threshold 0.8	normmi	*trilinear,*<br>*threshold 0.5*<br>***threshold 0.6***<br>*threshold 0.7*	mean×1.0	 0.504396±0.167<br>**0.527425±0.165***<br>0.524683±0.164
		nn	mean×1.0	0.526951±0.164
**Step 3. Calculate black hole intensity threshold**				
**Cortex selection**	**Cost function**	**Interpolation of the T2 lesion mask**	***Calculate black hole intensity threshold***	**DSC±SD**

WM_{PVE} + GM_{PVE} + MNI, threshold 0.8	normmi	trilinear threshold 0.6	*mean×0.95*<br>***mean×1.0***<br>*mean×1.05*	0.487601±0.182<br>**0.527425±0.165***<br>0.486859±0.163
			median×0.95<br>*median×1.0*<br>*median×1.05*	0.484532±0.185<br>0.523699±0.165<br>0.483780±0.163

corratio = correlation ratio, DSC = mean Dice similarity coefficient, GM = gray matter, MNI = Montreal Neurological Institute standard brain, nn = nearest-neighbor, normmi = normalized mutual information, SD = standard deviation, WM = white matter, WM_{class}; GM_{class} = WM or GM voxels from "hard" segmentation of brain tissue into three classes, WM_{PVE}; GM_{PVE} = voxelwise maps of partial volume estimates for WM and GM, respectively. The * symbol indicates the best result for a specific step. Italic font highlights the parameter that is tested in each step. Note that mean DSC values are provided in this Supplementary Table to six decimals in order to allow the reader to appreciate small performance differences between different parameter settings.

Note that although the average Dice scores are all lower than in Table 2 and Supplementary Table 4 (because of occasional mismatches where gold standard black hole lesion mask voxels occur outside of co-registered PD/T2 lesion masks, leading to black hole voxels that by definition cannot be detected by the new method, thus lowering the average Dice scores), the optimization process results in the same conclusions at each step. The only exception is the "cortex selection" step, in which in this unmasked ground truth analysis a threshold on the summed PVE maps of 1.0 produces a very slightly higher mean Dice score than the threshold of 0.8 favored by the analysis in the main paper with masked ground truth labels (0.500579±0.162 versus 0.500441±0.162). Interestingly, the intermediate threshold value of 0.9 produces average Dice score lower than either 0.8 or 1.0: 0.500159±0.162. In light of this latter fact and because the difference with the 0.8 threshold result is so small, we have continued in the remainder of this "unmasked ground truth" optimization with the 0.8 threshold value, as indicated in the Table.
